# Deep-subwavelength Decoupling for MIMO Antennas in Mobile Handsets with Singular Medium

**DOI:** 10.1038/s41598-017-11281-2

**Published:** 2017-09-22

**Authors:** Su Xu, Ming Zhang, Huailin Wen, Jun Wang

**Affiliations:** Huawei Technologies Co., Ltd., Hangzhou, 310027 China

## Abstract

Decreasing the mutual coupling between Multi-input Multi-output (MIMO) antenna elements in a mobile handset and achieving a high data rate is a challenging topic as the 5^th^-generation (5G) communication age is coming. Conventional decoupling components for MIMO antennas have to be re-designed when the geometries or frequencies of antennas have any adjustment. In this paper, we report a novel metamaterial-based decoupling strategy for MIMO antennas in mobile handsets with wide applicability. The decoupling component is made of subwavelength metal/air layers, which can be treated as singular medium over a broad frequency band. The flexible applicable property of the decoupling strategy is verified with different antennas over different frequency bands with the same metamaterial decoupling element. Finally, 1/100-wavelength 10-dB isolation is demonstrated for a 24-element MIMO antenna in mobile handsets over the frequency band from 4.55 to 4.75 GHz.

## Introduction

The next-generation communication devices have to achieve high data rate in order to meet the requirement of the 5G communication and the MIMO technique is one of the promising candidates to achieve this target^1^. However, placing a number of MIMO antenna elements in the limited space with low mutual coupling is a critical challenge in practical mobile handset design^2^. Serving as a powerful impetus to improve the efficiency and throughout of the communication system, several decoupling techniques have attracted considerable interest in recent years. Besides the neutralization line^3–5^ and defected ground structure^6^, decoupling matrix method^7–12^ can provide an alternative powerful and effective route to solve the decoupling issue by providing a current or S-parameter transfer matrix between the antenna ports. Generally, the decoupling matrix can be obtained by theoretical calculation or optimization algorithms and then realized by lumped elements, transmission lines and other planar structures. The decoupling matrix method can be used in the frequency adjustment^8^, beam forming^9^, and impedance matching^10^ as well, when the mutual coupling reduction is achieved.

Looking into the physical principle of these techniques, they share the idea of controlling interacted electric current distribution based on the model of transmission line. Generally, a specific decoupling structure designed from these current-based strategies can be only used for one or a few discrete frequency bands because the electric current distribution is highly frequency-dependent. It would be desirable for industrial applications if there were any alternative universal decoupling technique, which can achieve high isolation for different scenarios or different frequency bands with the use of a single geometrical design.

It is interesting that the two electrically small antenna elements with strong free-space coupling can be treated as two point sources with a deep subwavelength distance as they have several similarities. Firstly, both the geometrical dimensions of elements in two cases are in the subwavelength scale. Secondly, the distance between the antenna elements or point sources is less than 0.5 wavelengths. Thirdly, the free-space coupling will deteriorate the performance of the MIMO antennas, which is very similar to the evanescent wave decaying and subwavelength information loss during the imaging process. Therefore, the subwavelength imaging ideas may extend to the isolation design for MIMO antenna to solve the free-space coupling issue as we could make an analogy between the antenna elements with deep subwavelength distance in a mobile handset and discrete point sources limited by the diffraction limit.

The resolution of point sources is fundamentally limited to the diffraction limit^13^ as evanescent waves, which carry subwavelength information, decay exponentially in free space. With the development of metamaterial, perfect lens^14–19^ and hyperlens^20,21^ are able to recover subwavelength information carried by the evanescent waves with the use of metamaterials with negative parameters. Different from the negative-parameter-based subwavelength imaging techniques, canalization regime^22–26^ can also recover the subwavelength details of objects without the use of negative constitutive parameters by implementing the lens with metallic rod arrays as well^27–29^. Recently, Transformation Optics (TO) method^30,31^ presents potential ability in realizing deep subwavelength imaging over a broad frequency band^32–35^. The material used in the TO-based subwavelength imaging devices can be singular medium^36–41^, whose components of the permittivity or permeability tensors can be infinite or almost zero.

In this Letter, we report a TO-based practical broadband decoupling component for MIMO antennas in mobile handsets. The decoupling component is composed by alternate metallic and dielectric layers, which can be treated as singular medium. The effective singular medium exhibits highly anisotropy, little dispersion and lossless property, which would not decrease the efficiency of the MIMO antenna elements and can improve isolation over a broad frequency range. To study the extensive suitability, the metamaterial-based decoupling component with the identical geometry is verified with different antennas over different frequency bands. Finally, 1/100-wavelength 10-dB isolation is demonstrated for a 24-element MIMO antenna in mobile handsets over the frequency band from 4.55–4.75 GHz.

## Results

**Figure 1:**
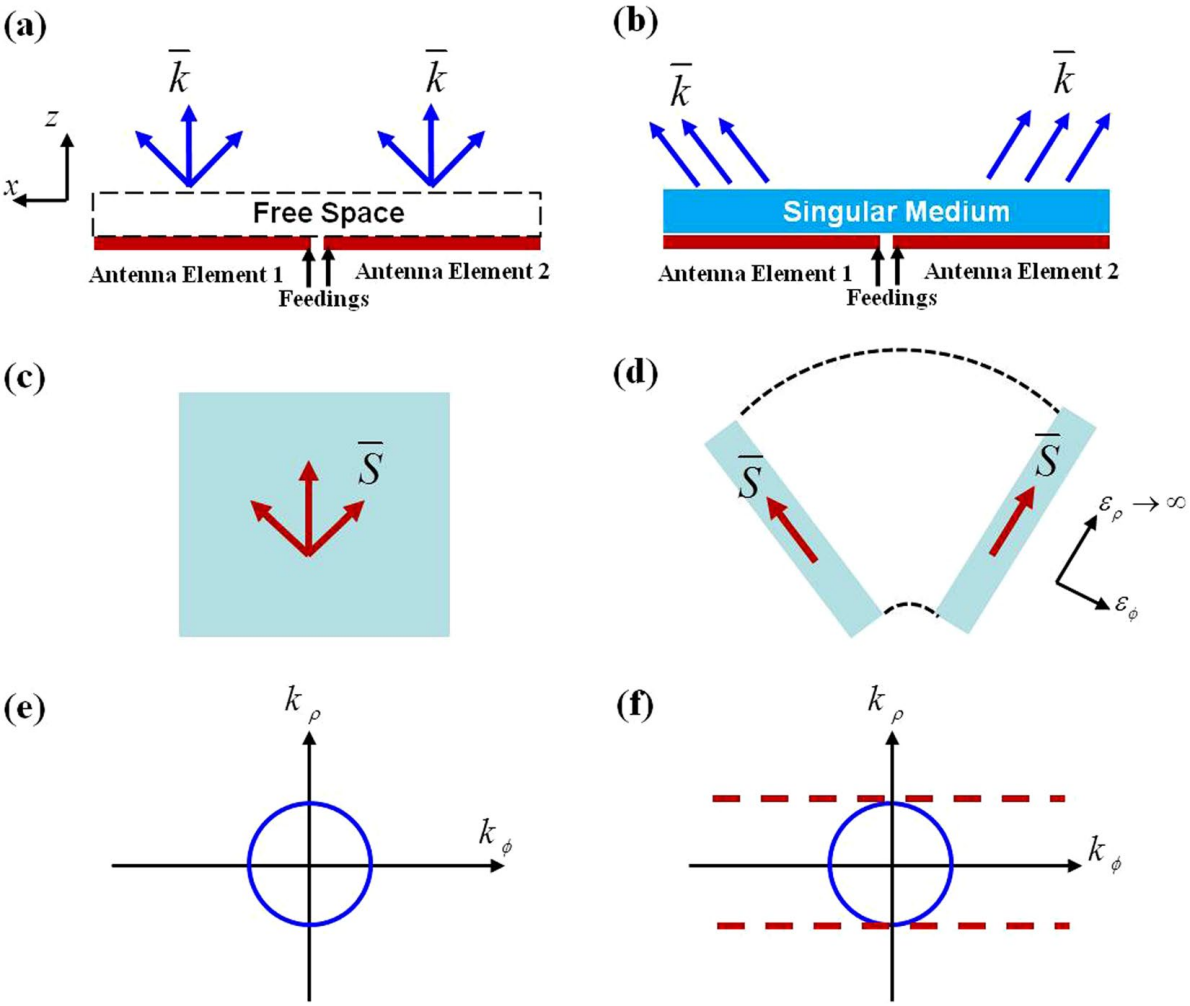
(**a**,**b**) The 2D scheme of a pair of antennas, (**c**,**d**) the corresponding virtual space and (**e**,**f**) *k* surfaces for the case without/with TO based decoupling component are illustrated here. Figs (**a**,**c**) and (**e**) are for the case without decoupling component, while Figs (**b**,**d**) and (**f**) are for the case with decoupling component. Only TM waves are considered.

Firstly we would like to introduce the basic physical principle of the singular medium-based decoupling structure briefly. The schematic of a pair of MIMO antenna elements without considering any decoupling recipe is shown in Fig. 1(a) and only transverse magnetic (TM) waves are considered here. Two antenna elements have to be located closely with a deep subwavelength distance as the available space for MIMO antenna elements in a mobile handset is limited. The dispersion of radiated electromagnetic waves without singular medium can be written as ${k}_{\rho }^{2}/{\varepsilon }_{\varphi }+{k}_{\varphi }^{2}/{\varepsilon }_{\rho }={\omega }^{2}{\mu }_{z}$ in cylindrical coordinate system, where *k*_*ρ*_ and *k*_*ϕ*_ are wave vectors along the radial direction *ρ* and azimuth direction *ϕ*, respectively. Similarly, *ε*_*ϕ*_ and *ε*_*ρ*_ present the permittivity coefficients along the *ϕ* and *ρ* directions, while *μ*_*z*_ presents the permeability coefficients along *z* direction. As shown in Fig. 1(c), the energy flows of electromagnetic waves $\bar{S}$, might spread out and the interaction between different antenna elements occurs. In this case, the *k* surface for free space (blue curve in Fig. 1(e)) is in a circular shape when there is no decoupling component.

To overcome the free-space mutual coupling induced by diffraction and loss of evanescent waves, a linear coordinate transformation procedure, i.e. $\rho ^{\prime} =\rho ,\varphi ^{\prime} =1/{N}^{\varphi }$, and *z*′ = *z*, is adopted in the design. Following this transformation procedure, the radial coefficient of relative permittivity becomes infinite as the squeezing factor *N* tends to infinity and then the dispersion relation become ${k}_{\rho }^{2}/{\varepsilon }_{\varphi }^{^{\prime} }={\omega }^{2}{\mu }_{z}$ when the magnetic field is perpendicular to the *ρϕ* plane. The radiated waves of antenna elements can only propagate along the radial direction and the energy flow is confined in the tube-like virtual spaces as shown in Fig. 1(d). The dispersion relation of transformed space is a pair of parallel lines as shown in Fig. 1(f), which can be realized with a singular medium. With such procedures, using a singular medium rather than any negative-index material can reduce the interacted radiation pattern of the MIMO antenna elements.

**Figure 2:**
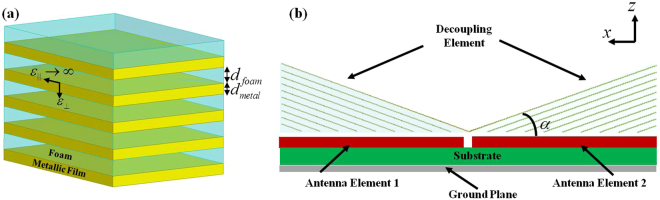
(**a**) The 3D schematic of layered structure composed of metallic films and foams. The deep-subwavelength periodicity *d* = *d*_foam_ + *d*_metal_, where *d*_foam_ and *d*_metal_ are the thicknesses of foam and metallic films, respectively. (**b**) The printed planar antenna pair with decoupling component. The decoupling component is formed by two triangular layered structures with an oblique angle *α*. The green layer, red layer and gray layer represent the dielectric substrate of the printed circuit board, the low-dielectric support/isolator (e.g. foam) between the decoupling component and the antennas, and the ground plane. The printed planar antenna pair is placed on the topside of the substrate.

As every coefficient of constitutive parameters can be positive in a singular medium, it is possible to propose a wideband decoupling component for MIMO antennas in mobile handset as long as the singular medium is realized without resonant structures. As shown schematically in Fig. 2, alternate metallic film and foam plates with subwavelength period can implement the singular medium. Based on the effective medium theory, the effective permittivity of this equivalent singular medium structure can be estimated as follows:

1 $${\varepsilon }_{\varphi }^{^{\prime} }={\varepsilon }_{\perp }=({\varepsilon }_{metal}{\varepsilon }_{foam})/(F{\varepsilon }_{foam}+(1+F){\varepsilon }_{metal}).$$

2 $${\varepsilon }_{\rho }^{^{\prime} }={\varepsilon }_{\parallel }=F{\varepsilon }_{metal}+(1-F){\varepsilon }_{foam},$$

where *F* = *d*_*metal*_/(*d*_*metal*_ + *d*_*foam*_) is the filling factor of the metal, *ε*_*metal*_ is the permittivity of metallic film, and *ε*_*foam*_ is the permittivity of foam with low dielectric constant. With Eqs (1 and 2), the effective permittivity of the layered structure can be expressed as${\varepsilon }_{\varphi }^{^{\prime} }={\varepsilon }_{\perp }=1/(1-F)$ and ${\varepsilon }_{\rho }^{^{\prime} }={\varepsilon }_{\parallel }\to \infty$ under the condition of *F* is far smaller than 1. The effective constitutive parameters indicate the layered structure can be treated as a singular medium and the decoupling component can be formed by two triangular layered structures, which placed with an oblique angle *α* between the surface of layered structure and the ground plane.

**Figure 3:**
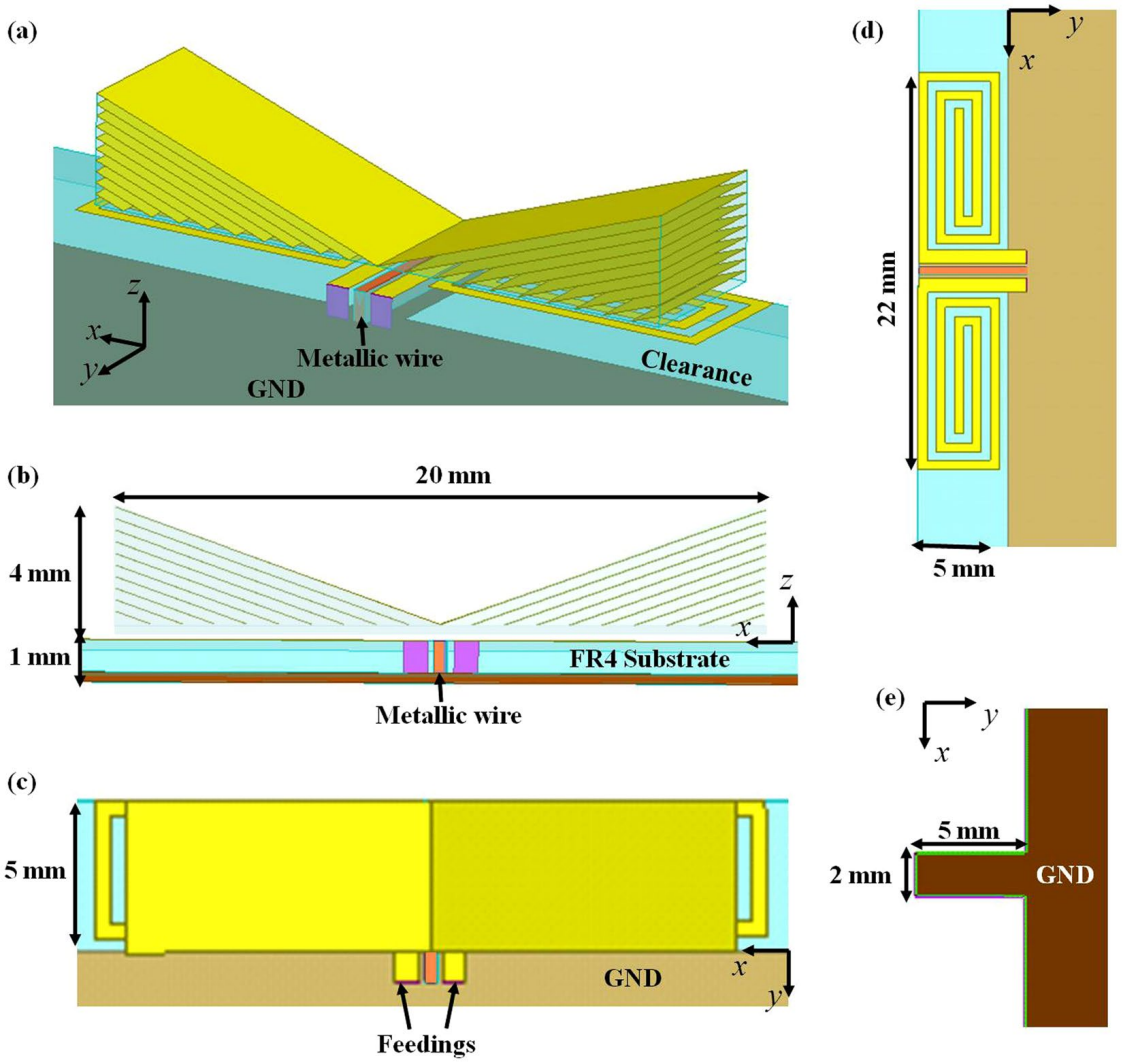
The monopole pair with the decoupling component: (**a**) 3D schematic of the whole structure, (**b**) front view, (**c**) top view with decoupling component, (**d**) top view without decoupling component, and (**e**) metallic strip and clearance on GND. The geometries of the decoupling element, antenna and the clearance are noted in the figure.

We firstly verify the performance of the decoupling component with a pair of planar monopole antennas over 4.55–4.75 GHz. Figure 3(a) shows the 3D schematic of the monopole antennas and decoupling component, while the dimensions of the monopole antennas and decoupling component can be found in Fig. 3(b–e). The monopole pair is placed on the top layer of a 1 mm-thick FR4 substrate while there is a 5 mm-wide clearance with a 2 mm * 5 mm metallic strip on the ground plane (GND). The distance between two feeding points of the planar monopole antennas is 0.8 mm (e.g. about 0.01 wavelengths at 4.65 GHz). The dimensions of the whole structure are 22 mm * 5 mm while the dimension of a single planar monopole antenna is 10.6 mm * 5 mm. Two triangular layered structures with the oblique angle of 22.6 degree make up one decoupling component with the dimensions of 20 mm * 5 mm * 4 mm. There is a 1 mm-height square metallic wire inserted in the substrate between two antennas to ensure the energy radiating into the free space rather than scattering into the substrate. To achieve the effect singular medium, the layered structure can be implemented periodically by 0.03 mm-thick aluminum foils and 1 mm-thick Rohacell^®^ HF71 foam with the relative permittivity of 1.10 at 10 GHz. According to Eqs (1 and 2), the effective permittivity coefficients are *ε*_*ϕ*_ = *ε*_⊥_ ≈ 1.1 and *ε*_*ρ*_ = *ε*_||_ → ∞. The infinite radial permittivity coefficient ensures the antenna radiation energy would not spread out and the mutual spatial coupling can be reduced, while the near-unity azimuth permittivity coefficient ensures the impedance matching at the antenna-decoupling component interface and decoupling component-free space interface. Although the squeezing factor should be a definite constant for a real practical design, the effective radial coefficient of permittivity can still tend to infinite because metallic films can be treated as perfect electric conductors at microwave regime.

**Figure 4:**
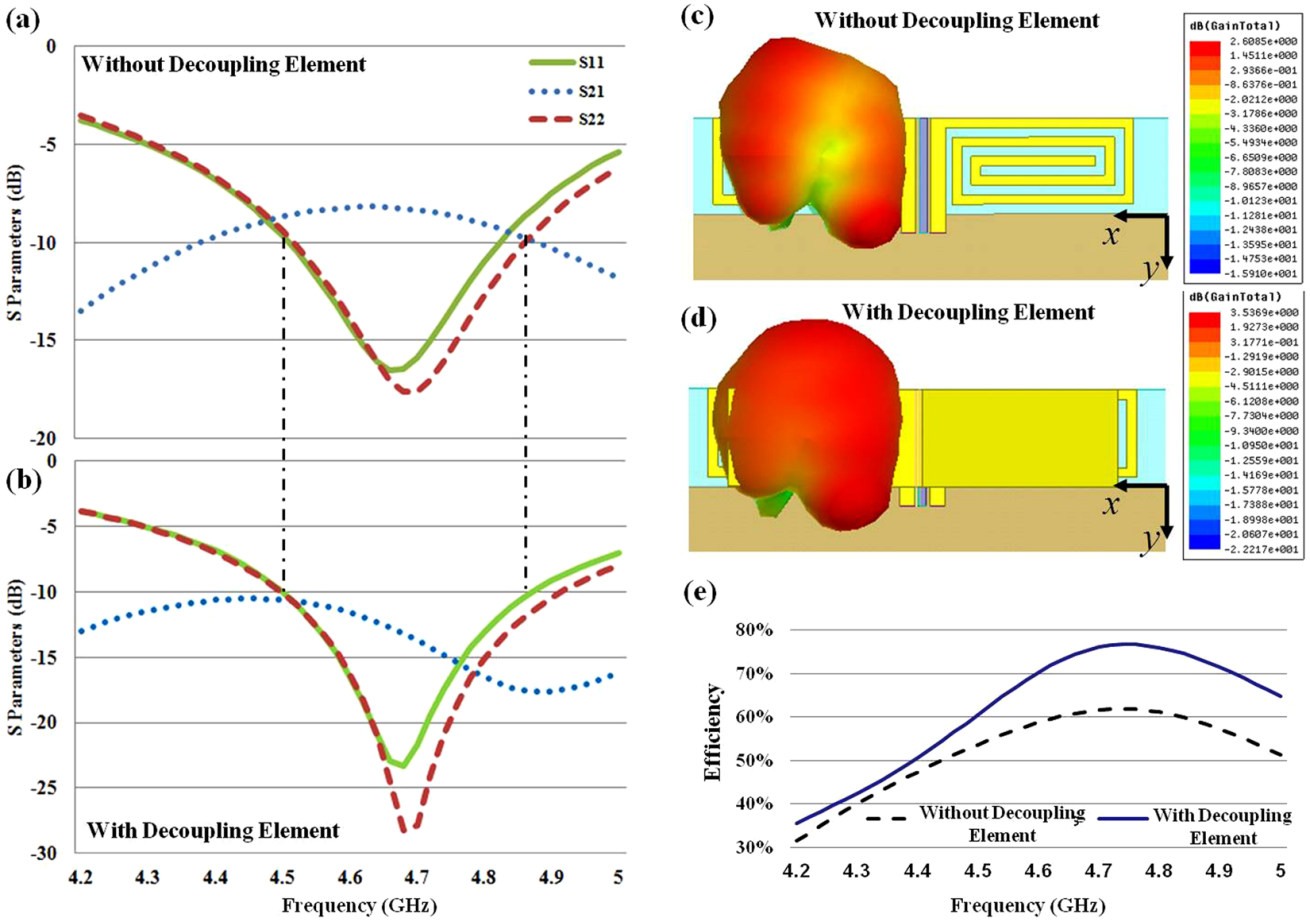
The S parameters of the monopole antenna pair for (**a**) the case without decoupling component and (**b**) the case with decoupling component are presented. The mutual coupling (dotted line in blue) can be decreased to a −10-dB level while the bandwidth of −10-dB return loss changed little. The radiation pattern for the cases (**c**) without and (**d**) with the decoupling component are compared, from which one can see the radiation pattern can be reshaped with the use of singular media. (**e**) The efficiency comparison with/without the decoupling component.

The performance of this decoupling component is studied with the use of the commercial software HFSS 15.0. Figure 4(a,b) shows the S parameters of the monopole pair without/with the decoupling component. For the case without decoupling component, the mutual coupling between two monopole elements is higher than −10 dB over the interested frequency band from 4.55–4.75 GHz. However, for the case with the decoupling component, the mutual coupling can be decreased to −10 dB over the whole interested frequency band. Because the antenna pair is not located at the midpoint of the ground edge, the asymmetric ground plane would introduce different surface current in the simulation. This will make the return loss of each antenna element different slightly even if the two antenna radiators have the same geometrical dimensions. Figure 4(c) and (d) show the radiation pattern of the antennas without/with the decoupling component. Compared to the case without decoupling component, we can see that the gain of antenna increases by 1 dB and the antenna directivity is improved in far-field region after being reshaped with the decoupling component. We also investigate the efficiencies with/without the decoupling component. As shown in Fig. 4(e), the efficiency of decoupled monopole antenna is 15% higher than the efficiency for case without the decoupling component.

**Figure 5:**
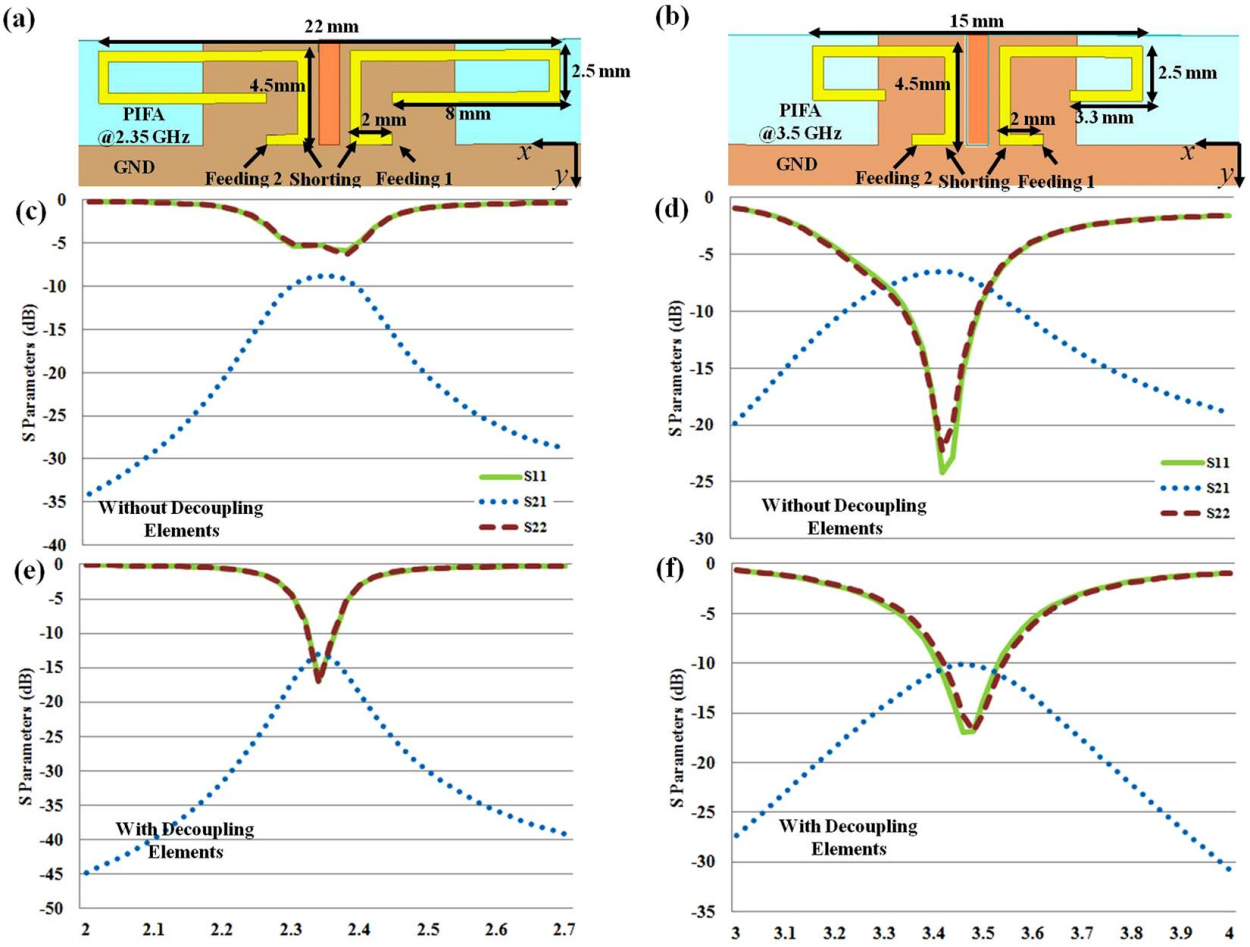
Two different PIFA with different working frequencies are studied to show the flexible applicability of our decoupling structure. Enclosed in the left column are the results of 2.35 GHz PIFA while the results of 3.5 GHz PIFA can be found in the right column. (**a**,**b**) The geometries of the PIFA antennas. (**c**,**d**) The S Parameters for the case without decoupling components. (**e**,**f**) The S parameters for the case with decoupling components.

It is interesting that the return loss of the antennas does not change much due to the wideband impedance matching between the antenna and the decoupling component. Therefore, it is possible to decrease the coupling for different types of antennas over different frequency bands. Two additional antennas are adopted here to study the flexible applicability. One antenna is a Planar Invert F Antenna (PIFA) radiating at the working frequency of 2.35 GHz (Fig. 5(a)), while the other one is a PIFA radiating at the working frequency of 3.5 GHz (Fig. 5(b)). The distance between two feeding pins of two PIFA elements is 2 mm (0.016 wavelengths at 2.35 GHz and 0.023 wavelengths at 3.5 GHz, respectively). Both the dimensions and the materials of the decoupling structure for PIFAs are identical to the one shown in Fig. 3. Including the case of the monopole antenna shown in Fig. 3, we totally have two types of antennas (monopoles and PIFAs) for three different frequencies to verify the wide applicable range of the decoupling component. Figure 5(c) and (e) shows the S parameters of the PIFAs for 2.35 GHz without/with the decoupling component. For the case without the decoupling component, both the mutual coupling and return loss are higher than −10 dB, which will deteriorate the performance of the antennas. However, the mutual coupling can be reduced to −13 dB and return loss can meet the −10 dB standard for the case with decoupling component. Similarly, the S parameters of the PIFAs for 3.5 GHz are shown in Fig. 5(d) and (f). For the case without the decoupling component, the mutual coupling is as high as −6 dB, whereas the mutual coupling can be decreased to −10 dB for the case with the decoupling component. Moreover, the bandwidths of the antennas change little because the azimuth coefficient of relative permittivity is close to unity in the decoupling component and the impedance between interfaces matches, which is an extremely desirable property for practical antenna with modular design in industrial context.

**Figure 6:**
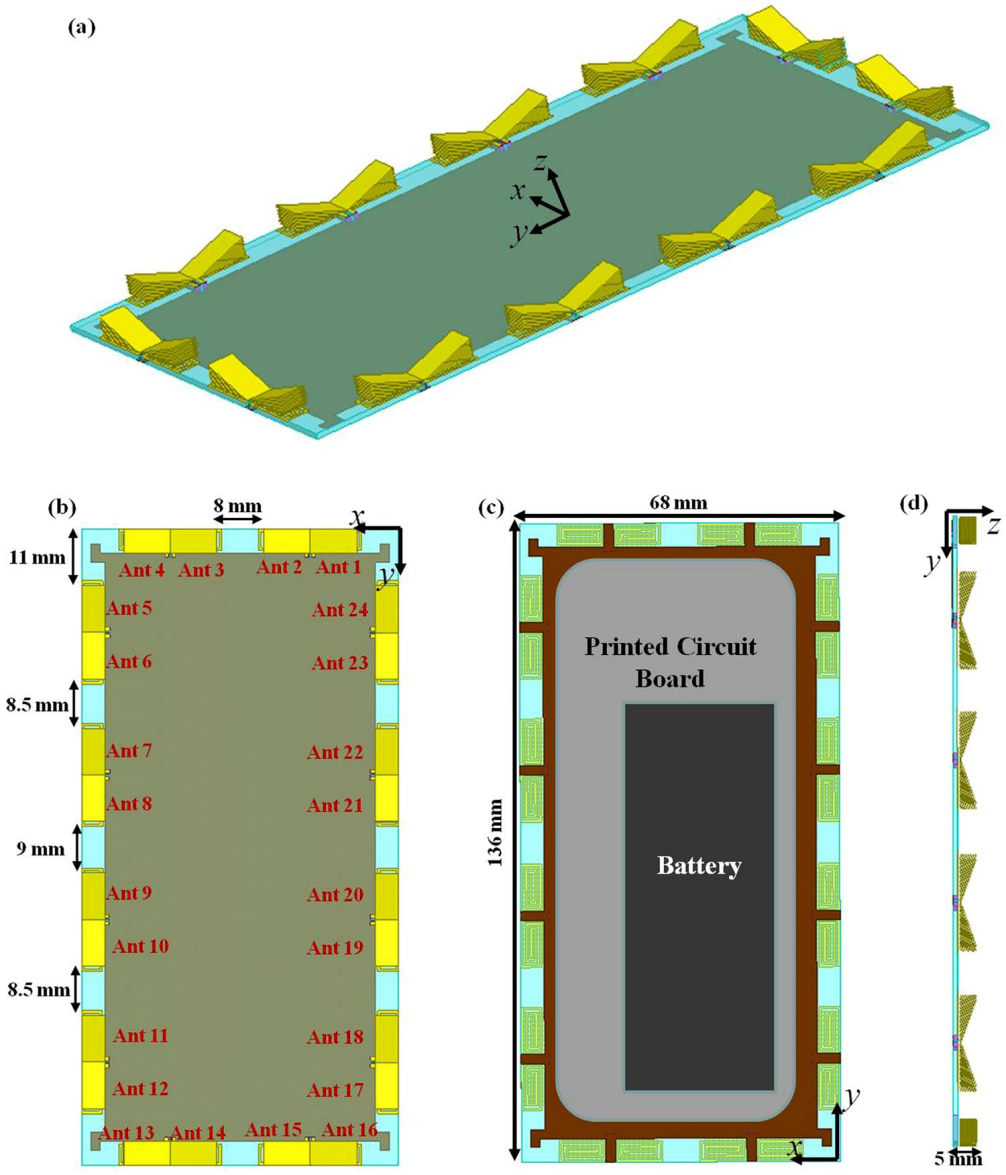
(**a**) Three dimensional schematic, (**b**) the top view, (**c**) bottom view and (**d**) side view of the 24-element MIMO antenna for mobile handset are illustrated here. The total geometrical dimensions are 136 mm * 68 mm * 5 mm. All the antenna elements are identical to the monopole antenna shown in Fig. 3.

**Figure 7:**
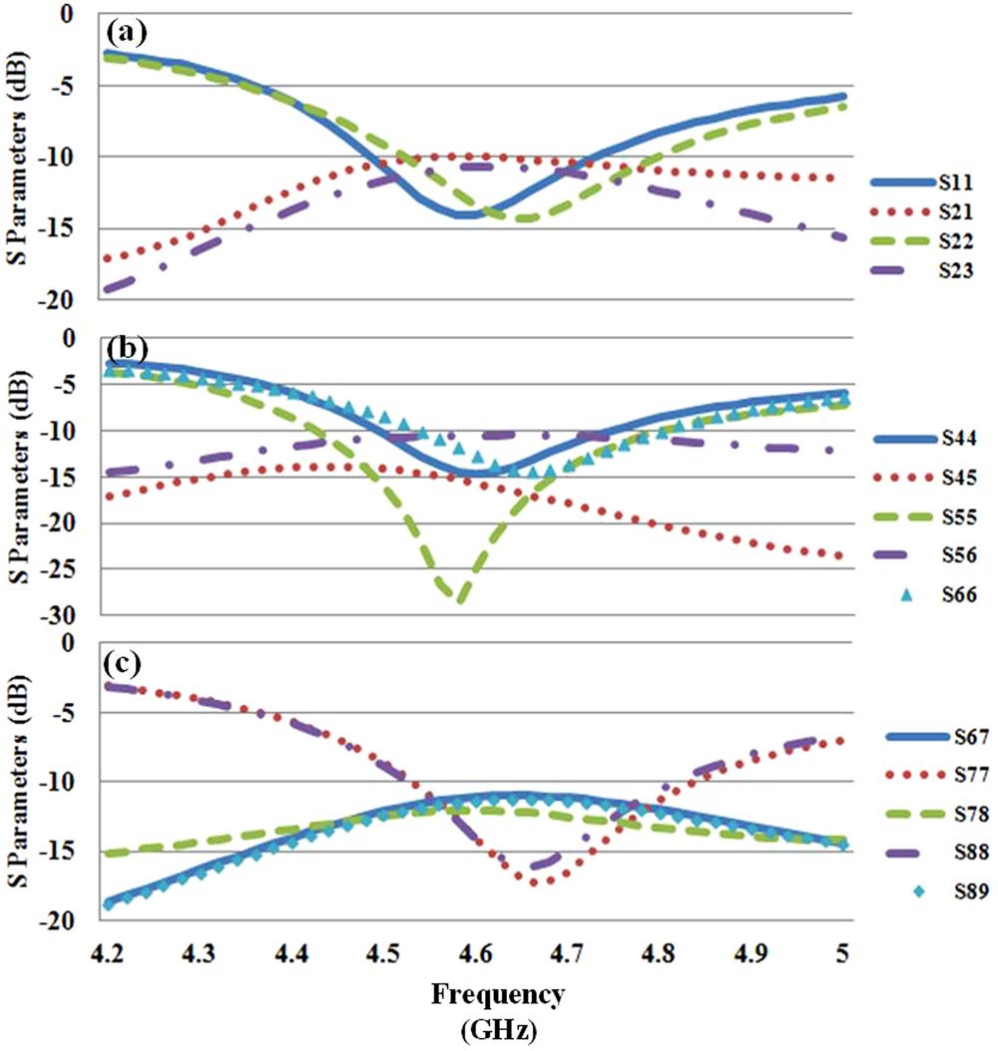
The S Parameters of the 24-element MIMO antenna: (**a**) Antennas on the short edge, (**b**) antennas around the corner and (**c**) antennas on the long edge of the substrate. Only the S parameters of selected antenna elements are illustrated here due to the symmetrical arrangement.

To explore the applicable maximum number of antenna elements in a single mobile handset, we design a 24-element MIMO antenna on a 1 mm-thickness FR4 substrate with the length of 136 mm and width of 68mm, which is usually used in the mobile phone design. The monopole antenna shown in Fig. 3 is used as the MIMO antenna unit cell in the design. The arrangement of the 24-element MIMO antenna is shown in Fig. 6 and all the antenna elements are placed at the edge of the substrate, as most of the areas on the board are for other electronic components (e.g. microphone, battery and printed circuit board). In the figure, “Ant *n*” represents the *n*th antenna element. Figure 7 shows the Simulated S parameters of the 24-element MIMO antenna. The return loss and mutual coupling of the antennas on the short edge, around the corner and on the long edge of the substrate are presented in Fig. 7(a–c), respectively. From the simulation results, we can see that the return loss and mutual coupling meet the −10 dB requirement for all the antenna elements over the whole interested frequency range from 4.55 GHz to 4.75 GHz. This design indicates that it is possible to place MIMO antenna elements on the edge of the small mobile handset for the 5G application. Moreover, we realized a MIMO antenna design, which involve the maximum number of MIMO element in a single mobile handset at the sub-6 GHz band, to the best of our knowledge.

## Discussion

As we introduced in the previous section, this decoupling structure can be used for different types of antennas over different frequency bands without changing any property of the decoupling structure. Due to the wide applicability on decreasing mutual coupling, our work provides an alternative way to achieve 5G MIMO applications for mobile handsets, which exhibits several differences compared to the decoupling matrix method. Firstly, these two methods involve different physical principles. Our work is based on the idea of controlling electromagnetic waves in the space, while the decoupling matrix method mainly operates the amplitude and phase of the current at the antenna ports. Secondly, the different physical principles introduce the different implementation techniques. For example, our work requires bulky novel electromagnetic materials, while the LC elements and transmission lines are usually used to form the decoupling matrix network. Finally, our work can be used for decoupling application over ultra-wide frequency band due to the dispersion-free property. As mentioned in the paper, the isolation for different antennas and different frequency bands can be improved without changing any property of the singular-medium structure. Decoupling matrix method can be easily adopted for decoupling application over narrow or single frequency band, but the calculation and realization complexity would increase greatly if we want to design a decoupling matrix network over an ultra wide frequency range. Therefore, the decoupling matrix method can decrease the mutual coupling efficiently in one or a few discrete narrow frequency bands, while our method have the potential ability to improve the isolation more easily over a continuous ultra wide frequency band.

There are some practical limitations relating to the bandwidth and applicable scenarios of our design. The first one is the theoretical model used in the design. According to the effective medium theory (EMT), the periodicity of the layered structure should be much smaller than the wavelength of interest (e.g. smaller than 1/5 wavelength). For instance, the 1-mm periodicity of layered structure used in this paper corresponds to 1/5 wavelength of 60 GHz, which indicate the structure can cover almost all the communication bands from the Long Term Evolution (LTE) to millimeter waves. The second limitation is about the applicable scenario. Because the TO method can manipulate the electromagnetic waves in free space or other bulk background materials, this technique is a powerful means of reducing the spatial coupling of radiated energy. However, it is difficult to control the interacted current flow on ground plane with a 3D singular medium hung over the planar printed or low-profile antennas. Fortunately as 2D metamaterials and surface wave manipulating strategies develop rapidly, there is possibility to find a quasi-universal 2D decoupling structure with a solution of manipulating electromagnetic waves in free space and surface wave/current simultaneously in future. For 5G applications, a large number of antenna elements should be arranged in the mobile handset, which introduces great challenges in decreasing mutual coupling between antenna elements. With our experience on antenna measurements, the mutual coupling shows little negative influence on antenna efficiency if 10-dB isolation can be achieved. That is why our goal is to improve the isolation to a 10-dB level in this work. This improvement is very helpful and effective for practical 5G MIMO applications in a mobile handset. The third point is about cost and complexity. Although the cost and complexity are important factors in commercial consideration, the principal challenges in practices are mainly about how to arrange enough number of antennas and how to achieve enough efficiency in the limited space of a single device at this stage of the 5G MIMO study for mobile handsets especially over the sub-6 GHz bands. Our work in this paper provides an alternative way to realize 5G MIMO applications for mobile handsets. It is our first step of our study on metamaterial-based 5G MIMO antennas for mobile handsets and we only pay attention to improve the isolation and efficiency now, rather than to suppress the cost and complexity. In the near future, we are going to push our work to a more practical case, such as integrating low-cost MIMO antennas with printed circuit board, battery and screen together.

In conclusion, we introduce a broadband decoupling component for MIMO antennas based on transformation optics. The decoupling component is made of alternate metallic and foam layers, which can be treated as singular medium. As the proof of concept, 1/100-wavelength 10-dB isolation is demonstrated for a 24-element MIMO antenna for mobile handsets over the frequency band from 4.55 to 4.75 GHz. The two significant advantages of this metamaterial-based decoupling structure are wide range of usability and inherent broadband phase matching at the interfaces for different antennas over different frequency bands. Therefore, this decoupling strategy can be treated as a quasi-universal decoupling solution and our work provides an alternative way for future practical modular design for antenna decoupling component in modern mobile handset industry.
